# Prevalence of depression and association with quality-of-life among oncology patients at Princess Marina Hospital, Botswana: A cross-sectional study

**DOI:** 10.4102/sajpsychiatry.v32i0.2586

**Published:** 2026-03-03

**Authors:** Swetha B. Jammalamadugu, Rebecca Luckett, Philip Opondo

**Affiliations:** 1 Department of Psychiatry, Faculty of Medicine, University of Botswana, Gaborone, Botswana; 2 Botswana Harvard Health Partnership, Gaborone, Botswana; 3 Department of Obstetrics and Gynaecology, Faculty of Medicine, University of Botswana, Gaborone, Botswana; 4 Department of Obstetrics & Gynecology, Beth Israel Deaconess Medical Center, Boston, United States

**Keywords:** cancer, depression, mental illness, oncology, quality-of-life, PHQ-9, WHO-BREF, prevalence

## Abstract

**Background:**

Depression significantly impacts cancer patients globally, complicating treatment outcomes by negatively affecting quality-of-life (QOL), self-care and treatment adherence. However, little is known about the prevalence of depression and its effects on QOL among cancer patients in Botswana.

**Aim:**

This study aimed to assess the prevalence of depression and its relationship with QOL among cancer patients at Princess Marina Hospital’s oncology unit.

**Setting:**

Patients seen at the oncology unit of Princess Marina Hospital, Botswana’s largest tertiary referral hospital.

**Methods:**

A cross-sectional study was conducted from February 2023 to August 2024. The sample size was 302. Socio-demographic data were collected using a researcher-designed questionnaire. Depression was assessed using the PHQ-9, with a score of 9 or higher indicating depression, and QOL was measured using the WHOQOL-BREF.

**Results:**

The prevalence of depression was 35.8% (95% confidence interval [CI]: 30.3%, 41.5%). Patients with depression had a higher mean pain score (6 vs. 4, *p* < 0.001) and poorer QOL across all domains: physical health (40.9 vs. 59.7, *p* < 0.001), psychological health (52.3 vs. 69.3, *p* < 0.001), social relationships (49.0 vs. 68.3, *p* < 0.001) and environmental health (48.1 vs. 58.8, *p* < 0.001). A new diagnosis of cancer was associated with an increased risk of depression (*p* = 0.03).

**Conclusion:**

The prevalence of depression among cancer patients is high in Botswana, and it is associated with poorer QOL, highlighting a significant public health concern.

**Contribution:**

Treatment of depression in this group of patients, along with pain management, may be crucial in improving their QOL.

## Introduction

In 2022, approximately 19.9 million new cancer cases were documented globally, with projections of 27 million new cases by 2030.^1,2^ Cancer incidence rates in high-income countries (HICs) are stabilising and even decreasing.^3^ Despite HICs having a higher burden of cancer, their mortality rates are lower relative to incidence. In contrast, lower middle-income countries (LMICs) typically have lower cancer incidence reported but often experience higher mortality rates relative to incidence.^3^ Over 70% of cancer fatalities occur in LMICs,^4^ which may be a result of limitations in diagnosis and delays in work-up.^3^ In 2020, Botswana recorded around 2010 new cancer cases and approximately 1112 cancer-related deaths.^5^ While cancer is not the primary cause of death in Botswana, it is becoming a growing concern, accounting for 7% of all deaths.^6^

Cancer is a life-altering diagnosis and carries a significant psychological burden, increasing the risk of depression, impacting patients’ quality-of-life (QOL), self-care, immunity and treatment adherence.^7^ The prevalence of depression among cancer patients has been reported as variable across settings, ranging from 8% to 49%,^8^ with an average burden of 27%.^9^ Comorbid depression may arise from various physical factors such as uncontrolled pain, metabolic and endocrine disruptions.^10^ The psychological strain of cancer diagnoses and treatments,^5^ including fear of further morbidity and mortality, can lead to heightened depression among patients. Studies indicate a higher suicide risk even after curative treatments.^11^ Severe depression has been associated with a lower QOL and even increased mortality among cancer patients.^12^ Patients suffering from depression are often less likely to pursue recommended treatment options, such as chemotherapy, compared to their non-depressed counterparts.^13^ Yet, mental health disorders associated with cancer often go underdiagnosed as a result of a variety of factors, including lack of awareness about psychiatric symptoms, insufficient support from healthcare providers and the stigma surrounding mental health.^14^

Globally, depression is the leading cause of disability.^15^ More than 80% of the burden of depression is believed to lie within LMICs, yet only one in three cases is recognised as needing treatment.^16^ The prevalence of depression in Botswana is notably high at 30.4% as of 2020,^17^ significantly exceeding the global average of 5%^15^ and has been associated with chronic illnesses, such as HIV and diabetes. A study at Princess Marina Hospital found that 38% of patients living with HIV met the criteria for depression.^18^ Similarly, among diabetic patients, the prevalence of depression was reported at 30.4%.^19^

The prevalence of depression among cancer patients in Botswana remains under-explored and could significantly impact their survival rates and overall QOL. There are no studies that look at the mental health of oncology patients in Botswana. Understanding the burden of depression and factors associated with depression in cancer patients is essential for developing targeted interventions that improve both mental health and QOL, ultimately enhancing survival rates in this vulnerable population.^13^ This study aimed to assess the prevalence of depression and its relationship with QOL among cancer patients at Princess Marina Hospital’s oncology unit.

## Research methods and design

### Study setting

The study was conducted in the oncology in-patient ward and out-patient clinic at Princess Marina Hospital, the main cancer referral centre for the southern part of the country, which serves a diverse range of cancer patients. The out-patient clinic and in-patient unit are housed within the same building and clinical staff move between both units.

### Study design

This is a descriptive study that used a cross-sectional design. It was conducted from February 2023 to August 2024. Convenience sampling was employed for several reasons. The study was conducted as part of a training programme, which restricted what could be accomplished because of limited research time. In addition, in-patient turnover was limited because patients who lived far from the hospital needed to remain hospitalised for the duration of their treatment, including procedures typically conducted on an out-patient basis, such as chemotherapy infusions, and as a result, out-patient recruitment was higher than in-patient recruitment.

### Participants and procedures

To be precise, 302 oncology patients agreed to participate in this study. Any client with a diagnosis of cancer who was 21 years old or older and who was an in-patient or out-patient seen in the oncology unit at Princess Marina Hospital was included in the study. Patients who were too sick to answer the questions (e.g. those with altered mental status or who were being treated in the high dependency ward or who were acutely ill needing emergent medical attention) were excluded from the study.

In-patients and out-patients were recruited on high-volume clinic days when oncology physicians were seeing out-patients. A general announcement was made in the ward and in the out-patient waiting area, and those who showed interest were approached personally and provided information about the study. Those who were willing to participate provided verbal consent.

Consented participants completed a questionnaire in either Setswana or English, and assistance was provided by the study team for participants unable to complete it by themselves because of their level of education or physical disability.

The structured questionnaire collected self-reported demographic and medical history data, including age, gender, average income, level of education, comorbidities, history of mental health illness and family history of mental illness. The history of mental illness included any psychiatric diagnosis that the patient reported, including depression. This was a cross-sectional study that looked at the current mental state of the participants; therefore, previous diagnosis of depression before cancer diagnosis was recorded but not considered during analysis. Participants were asked to subjectively rate their current pain level on a pain scale from 1 to 10, where 1 was no pain and 10 was extreme pain (the worst pain of their life). Participants then completed the Patient Health Questionnaire-9 (PHQ-9) and the World Health Organization QOL scale Brief Version (WHO-QOL-BREF) questionnaires.

Sensitive topics were handled with utmost care and addressed in a confidential manner and with respect for the participants. Participants who exhibited signs of depression or suicidality, based on their answers to the PHQ-9 (i.e. self-report), were referred for appropriate care.

### Data collection and materials

All the instruments used in this study were translated and back-translated into Setswana by independent translators to ensure accuracy and quality.

The PHQ-9 is a freely available screening tool for depression that has been validated for use in Botswana.^20^ It is a tool that consists of nine screening questions, and each question is scored from 0 to 3 points with a maximum score of 27. High scores on the PHQ-9 indicated that at the time the questionnaire was administered, the participant had symptoms suggestive of depression. The WHO-QOL-BREF tool measures various subjective aspects of QOL including physical health, psychological health, social relationships and environmental factors that lead to decreased QOL. Each of 20 questions is scored on a Likert scale from 1 to 5, and the overall score (out of 100) grades the QOL of the individual.^21^ The maximum score is 100, and the minimum score is 0, where the higher the score, the better the perceived QOL. In similar studies, the internal consistency was high for all domains of the WHO-QOL-BREF (Cronbach’s α ≥ 0.731).^22^ In this study, Cronbach’s α = 0.8, suggesting that the internal consistency of each part of the WHO-QOL-BREF was good.

### Data analysis and sample size calculation

The primary outcome was the prevalence of depression in the population of cancer patients at Princess Marina Hospital. The secondary outcomes were factors associated with depression and QOL. Data were collected on Microsoft Excel and exported for data analysis to Statistical Package for Social Sciences (SPSS for Windows 29.0.2.0 SPSS Inc., Chicago, IL, United States) and Stata Statistical Software (StataCorp. Stata Statistical Software: Release 15. College Station, TX: StataCorp LP; 2017). Descriptive statistics were presented in frequency tables and charts. Categorical variables were expressed in proportions or percentages. Continuous variables were summarised by means and standard deviation or medians and interquartile range. T-test was used for comparing continuous variables, while categorical variables were assessed by the chi-square test. Depression was diagnosed using a PHQ-9 score of 9 or higher, based on a meta-analysis indicating that this cut-off offers optimal sensitivity and specificity.^23^ This cut-off was used in the dichotomous analysis, and in addition, because the severity of depression is directly proportional to the PHQ-9 score, the score was also analysed as a continuous variable. The WHO-QOL-BREF score was considered a continuous variable where scores closer to 0 were indicative of poor QOL and those closer to 100 indicated good QOL. Missing demographic data were noted during analysis and indicated in the tables where relevant.

A Poisson regression model with robust standard errors was used to estimate the association between demographic and clinical factors and the prevalence of depression. This model was chosen because the prevalence of depression in our study population was 35.8%, which exceeds 10%, making the outcome common; in such cases, prevalence ratios (PR) provided by Poisson regression are more accurate and interpretable than odds ratios (ORs). Univariable and multivariable analyses were performed, respectively. In the univariable analysis, each factor was tested individually to determine its association with depression. Following that, a multivariable model was constructed using a forced-entry (a priori) approach, including all collected demographic and clinical variables regardless of their univariable significance. This was done to rigorously control for potential confounding and to determine the independent effect of each predictor, which is represented as the adjusted prevalence ratio (aPR).

Based on prior literature, we predicted a prevalence of 25% for depression among cancer patients,^24^ and thus we required a minimum sample size of 288 participants with a standard deviation of 1.96 for a 95% confidence interval and a degree of error estimated at 5%.

### Ethical considerations

This study followed the principles outlined by the Declaration of Helsinki. Ethical clearance to conduct this study was obtained from the Institutional Review Board of University in Botswana, the Ministry of Health and Wellness and Princess Marina Hospital (Ref. No. UBR/RES/IRB/BIO/GRAD/170, HPDME: 13/8/1 and PMH 2/11AII [208]). Verbal consent was obtained from each of the participants. Data were collected, and a code was assigned to each participant. The link between the code and the participant’s details was kept in a separate place such that there was no bias during data entry and analysis. Verbal consent was chosen as a mode as the approval for this study was sought during the coronavirus disease 2019 (COVID-19) pandemic, necessitating a model that would keep the researchers and the participants safe.

## Results

### Socio-demographic factors and association with depression

To be precise, 302 oncology patients (28% in-patients and 72% out-patients) at Princess Marina Hospital were recruited and consented. The prevalence of depression among our study population was 35.8% (108) (95% confidence interval [CI]: 30.3%, 41.5%).

Socio-demographic characteristics (Table 1) were compared between participants with and without depression; however, no significant differences were found. The median age was 55 years (interquartile range [IQR] 43.5–66), and 71% were female. One-third of the participants were married, and the majority were Christians (92%). Most of the participants (210, 70%) were unemployed and therefore did not have a personal income; the remaining 92 participants reported an average income of $29 per month.

**TABLE 1:** Socio-demographic characteristics of participants with and without depression.

Variable	Total population (*N* = 302)	Depression (*N* = 108)		No depression (*N* = 194)		χ^2^	*P*-value
		*n*	%	*n*	%		
**Gender**						0.017	0.89
Male	101	37	36.6	64	63.4	-	-
Female	198	71	35.9	127	64.1	-	-
**Ethnicity**						0.538	0.75
Tswana	209	72	34.4	137	65.6	-	-
Kalanga	11	4	36.4	7	63.6	-	-
Other	82	32	39.0	50	61.0	-	-
**Marital status**						1.790	0.20
Married	102	31	30.4	71	69.6	-	-
Other†	199	76	38.2	123	61.8	-	-
**Employment**						0.001	0.99
Employed	92	33	35.9	59	64.1	-	-
Unemployed	210	75	35.7	135	64.3	-	-
**Level of education**						1.471	0.69
None	47	19	40.4	28	59.6	-	-
Primary	65	20	30.8	45	69.2	-	-
Secondary	108	41	38.0	67	62.0	-	-
Tertiary	82	28	34.1	54	65.9	-	-
**Religious affiliation**						1.342	0.28
Christian	277	102	36.8	175	63.2	-	-
Other	24	6	25.0	18	75.0	-	-
**Religious activity level**						0.967	0.82
None	24	8	33.3	16	66.7	-	-
Rarely	40	12	30.0	28	70.0	-	-
Sometimes	88	34	38.6	54	61.4	-	-
Regularly	140	49	35.0	91	65.0	-	-
**In-patient vs. out-patient**						0.483	0.51
In-patient	85	33	38.8	52	61.2	-	-
Out-patient	217	75	34.6	142	65.4	-	-

† , Single, In a relationship, Widowed, Divorced, Cohabiting.

### Medical characteristics

The subjective mean pain score reported by participants was 4 (± standard deviation [s.d.] 3), and participants with depression reported higher mean pain scores than those without depression (6 vs. 4, *p* < 0.001). Between those with and without depression, there were no differences in prevalence of chronic diseases, including HIV, personal history of mental illness and hypertension (*p*-value > 0.05). There was also no difference in family history of mental illness between those with and without depression (*p* = 0.378). While not statistically significant, a higher proportion of participants with type 2 diabetes were depressed (52.6%) compared to those without (34.6%) (Table 2).

**TABLE 2:** Prevalent chronic diseases and family mental health history among participants with and without depression.

Variable	Overall (*N* = 302)		Depression (*N* = 108)		No depression (*N* = 194)		χ^2^	*P*-value
	*n*	%	*n*	%	*n*	%		
**HIV status †**							0.19	0.91
Positive	115	38.3	41	35.7	74	64.0	-	-
Negative	172	57.3	63	36.6	109	63.4	-	-
Unknown	13	4.3	4	30.8	9	69.2	-	-
**Personal history of mental illness†**							0.09	0.92
Yes	3	1.0	1	33.3	2	66.7	-	-
No	297	99.0	107	36.0	190	64.0	-	-
**History of hypertension**							1.055	0.29
Yes	95	31.5	30	31.6	65	68.4	-	-
No	207	68.5	78		129		-	-
**Type 2 diabetes mellitus**							2.512	0.15
Yes	19	6.3	10	52.6	9	47.4	-	-
No	283	93.7	98	34.6	185	65.4	-	-
**Chronic kidney disease**							2.545	0.11
Yes	6	2.0	4	66.7	2	33.3	-	-
No	296	98.0	104	35.1	192	64.9	-	-
**History of stroke**							1.121	0.29
Yes	2	0.7	0	0.0	2	100.0	-	-
No	300	99.3	108	36.0	192	64.0	-	-
**Anaemia**							2.83	0.92
Yes	5	1.7	0	0.0	5	100.0	-	-
No	297	98.3	108	36.4	189	63.6	-	-
**Other chronic conditions**							1.89	0.17
Yes	20	6.6	10	50.0	10	50.0	-	-
No	282	93.4	98	34.8	184	65.2	-	-
**Family history of mental illness**							0.195	0.38
Yes	39	12.9	15	38.5	24	61.5	-	-
No	245	81.1	84	34.3	161	65.7	-	-

† , Missing data: HIV status 2, personal history of mental illness 2.

There was no statistically significant difference between the type of cancer and participants with depression (Table 3).

**TABLE 3:** Type of cancer among participants with and without depression.

Type of cancer	Overall (*N* = 302)		Depression (%) (*N* = 108)	No depression (%) (*N* = 194)
	*n*	%		
Breast	75	24.80	23.15	25.77
Gynaecological cancers	57	18.87	20.37	18.04
Uro genital tract	47	15.56	14.81	15.98
GIT	19	6.29	7.41	5.67
ENT	17	5.63	7.41	4.64
Blood	14	4.64	2.78	5.67
Sarcomas	13	4.30	3.70	4.64
Lymphoma	10	3.31	3.70	3.09
Skin	8	2.65	2.78	2.58
Brain and spinal cord	5	1.66	3.70	1.55
Lung	4	1.32	0.93	1.55
Bone	3	0.99	1.85	0.52
Other	2	0.66	0.93	0.52
Metastasised and/or location unclear	1	0.33	0.00	0.52
Thyroid	1	0.33	0.93	0.00
Unknown	26	8.61	7.41	9.28

Note: χ^2^ = 7.511; *p*-value = 0.94.

GIT, Gastrointestinal; ENT, Ear Nose and Throat.

In univariable analysis, none of the socio-demographic and clinical characteristics showed a statistically significant association with depression. Similarly, in the multivariable model adjusting for all covariates, most factors, including age, sex, ethnicity, marital status, occupation, education, religion, patient status, HIV status, hypertension and history of myocardial infarction, remained not significantly associated with depression (all *p* > 0.05).

In both univariable and multivariable models, participants with a new cancer diagnosis had a significantly higher prevalence of depression compared to those currently on treatment (aPR = 1.10; 95% CI: 1.01–1.19; *p* = 0.03). Participants in the palliative phase or post-treatment did not show statistically significant differences in depression prevalence compared to the reference group (palliative: aPR = 0.75; 95% CI: 0.51–1.10; *p* = 0.15; post-treatment: aPR = 0.99; 95% CI: 0.88–1.11; *p* = 0.89).

However, type 2 diabetes was a significant predictor of depression in the adjusted model. Participants with diabetes had a 16% lower prevalence of depression compared to non-diabetic participants (aPR = 0.84; 95% CI: 0.71–0.99; *p* = 0.033) (Table 4).

**TABLE 4:** Multivariate analysis of depression and its associated factors.

Variable (Category)	Univariable PR	95% CI	*P*-value	Multivariable aPR	95% CI	*P*-value
Age (years)	1.00	1.00–1.00	0.26	1.00	0.998–1.00	0.36
**Sex**						
Male	Ref	-	-	Ref	-	-
Female	1.00	0.94–1.08	0.9	1.01	0.93–1.09	0.85
**Marital status**						
Married	Ref	-	-	Ref	-	-
Other	0.95	0.89–1.02	0.17	0.97	0.90–1.04	0.4
**Occupation**						
Unemployed	Ref	-	-	Ref	-	-
Employed	1.00	0.93–1.07	0.98	1.02	0.94–1.11	0.68
**Education**						
None	Ref	-	-	Ref	-	-
Primary	1.06	0.95–1.18	0.3	1.07	0.94–1.22	0.32
Secondary	1.02	0.91–1.13	0.77	1.04	0.91–1.19	0.55
Tertiary	1.04	0.93–1.16	0.48	1.07	0.93–1.23	0.33
**Religion**						
Christian	Ref	-	-	Ref	-	-
Other	1.07	0.97–1.19	0.19	1.08	0.95–1.23	0.24
**Religious activity**						
Always	Ref	-	-	Ref	-	-
Often	0.99	0.88–1.12	0.87	1.05	0.90–1.23	0.52
Rarely	1.02	0.89–1.17	0.78	1.07	0.91–1.25	0.44
Sometimes	0.97	0.85–1.10	0.63	1.04	0.89–1.21	0.66
**Ethnicity**						
Other	Ref	-	-	Ref	-	-
Kalanga	0.99	0.85–1.15	0.85	0.99	0.84–1.17	0.91
Tswana	1.01	0.88–1.17	0.84	1.01	0.92–1.10	0.91
**Patient status**						
In-patient	Ref	-	-	Ref	-	-
Out-patient	1.03	0.95–1.11	0.5	1.00	0.91–1.10	0.98
**HIV status**						
Negative	Ref	-	-	Ref	-	-
Positive	1.01	0.94–1.08	0.87	1.01	0.93–1.10	0.85
Unknown	1.04	0.89–1.21	0.66	0.96	0.77–1.20	0.73
**Hypertension**						
No	Ref	-	-	Ref	-	-
Yes	0.96	0.90–1.03	0.29	1.04	0.96–1.14	0.31
**Type 2 diabetes**						
Non-diabetic	Ref	-	-	Ref	-	-
Diabetic	0.89	0.76–1.04	0.15	0.84	0.72–0.99	**0.04**
**History of MI**						
No	Ref	-	-	Ref	-	-
Yes	1.02	0.74–1.40	0.92	1.03	0.74–1.43	0.85
**Treatment status**						
Currently on treatment	Ref	-	-	Ref	-	-
New diagnosis	1.10	1.02–1.19	**0.01**	1.10	1.01–1.19	**0.03**
Post-treatment	1.02	0.92–1.14	0.67	0.99	0.88–1.11	0.89
Palliative	0.93	0.71–1.22	0.61	0.75	0.51–1.10	0.15

MI, Myocardial Infarction; CI, confidence interval; PR, prevalence ratio; aPR, adjusted prevalence ratio.

### Depression and quality-of-life

Depression was significantly associated with reduced QOL scores across all four domains, including physical health, psychological health, social relationships and environment (Table 5). There was a negative correlation between depression and the domains of physical health, psychological health, social relationships and environment (Table 6). In contrast, pain was positively correlated with depression scores, suggesting that higher pain levels are associated with increased depression scores, while the opposite is true for QOL scores.

**TABLE 5:** Mean scores of quality-of-life assessment of participants with and without depression.

Domain	Depression (*N* = 108)	No depression (*N* = 194)	Mean difference	95% CI	*P*-value
Physical health	40.9	59.7	−18.8	−23.5, -14.0	< 0.001
Psychological health	52.3	69.3	−17.0	−21.4, -12.6	< 0.001
Social relations	49.0	68.3	−19.3	−25.4, -13.2	< 0.001
Environment	48.1	58.8	−10.7	−15.3, -5.9	< 0.001

CI, confidence interval.

**TABLE 6:** Relationship between significant clinical variables and depression as continuous variables.

Variables	1	2	3	4	5	6	7	8
Pain scale	1.00	-0.13*	0.11	0.41**	-0.50**	-0.29**	-0.28**	-0.29**
Income per month (USD)	-	1.00	-0.09	0.09	0.10	0.04	0.01	0.22**
Age	-	-	1.00	-0.03	-0.03	0.07	-0.08	0.09
Depression	-	-	-	1.00	-0.51**	-0.50**	-0.36**	-0.29**
Physical health	-	-	-	-	1.00	0.60**	0.42**	0.53**
Psychological health	-	-	-	-	-	1.00	0.50**	0.57**
Social relations	-	-	-	-	-	-	1.00	0.41**
Environment	-	-	-	-	-	-	-	1.00

USD, United States Dollar

* , Correlation is significant at the 0.05 level (two tailed).

** , Correlation is significant at the 0.01 level (two tailed).

Pain was identified as a major factor associated with depression, affecting multiple aspects of QOL

## Discussion

This study sought to assess the prevalence of depression and its relationship with QOL in cancer patients at the oncology unit in the main tertiary referral centre in Botswana. We found a high rate of depression (35.8%) among the patients. The burden of depression among cancer patients in Botswana has not been well studied, yet depression may greatly affect not only overall QOL but also ultimately survival. The prevalence of depression among cancer patients in our population was supported by data from prior studies, closely aligning with the African average of 53.21%^25^ and the global estimate of 33.16%.^26^ A systematic review focusing on breast cancer patients revealed a global depression prevalence rate of 32.2%, with significantly higher rates in middle-income countries compared to high-income countries.^17^ In Nigeria, nearly 49% of cancer patients exhibited depressive symptoms, underscoring the mental health challenges faced by this population.^17^ Among patients with cervical cancer in Korea, a comparable depression rate of 39.5% was observed, a finding that is particularly relevant to our population in Botswana, where cervical cancer remains the leading cause of cancer in women.^27^ Although the study enrolled more women than men, the difference in depression prevalence between sexes was not statistically significant.^28^

While prior studies in Botswana showed high rates of depression among people with diabetes (30.4%),^19^ this study found, during multivariate analysis, that having diabetes was a protective factor against depression, which may suggest that patients with type 2 diabetes were less likely to have depression than those without depression, which is contrary to the current available evidence that suggests otherwise.^29^ The association of medical comorbidities, cancer and depression merits further exploration given our findings that cancer patients with diabetes had lower rates of depression and cancer patients with HIV have no increased risk for depression, both of which contradict prior literature on the association of those medical comorbidities with depression.^28^ Only 1% of participants reported a prior mental illness diagnosis, far below global estimates, likely highlighting the need for better recognition and management of depression in general care.

Multivariate analysis also showed that being newly diagnosed with cancer was a key factor linked to depression. This finding is in keeping with existing literature that shows that depression is most strongly associated with cancer in the initial year following diagnosis, with the association diminishing gradually as time passes.^30^

Psychological factors, such as demoralisation, have previously been correlated to depression,^31^ which supports findings in the current study where psychological aspects of QOL had a statistically significant, negative correlation with the presence of depression in this population. Prior research had found that as religious attitudes increased depression and hopelessness scores decreased^32^; however, this study did not find any such relationship, possibly because our population was a relatively homogenous group of fairly religiously active Christians.

Research in the United States (US) identified that better coping with depression in cancer patients is associated with factors such as pain relief, female sex, disease-free status and higher socio-economic status.^33^ Furthermore, a study involving Chinese oncology in-patients found a 25.9% prevalence of depressive disorders, with specific factors like marital status and cancer stage correlating with higher depression rates.^34^ However, this study did not find a significant relationship between female sex, socio-economic status and marital status.

In a study conducted in Ethiopia, 69.0% of participants with an established diagnosis of cancer reported pain. Individuals experiencing pain had over four times the likelihood of showing major depressive symptoms.^35^ A review of pain management and palliative care in Kenya and Uganda stated that pain relief (particularly opioids) is largely unavailable in the public health setting, contributing to poor pain management among cancer patients.^36^ In this study, pain was significantly associated with depression, suggesting that managing pain in this population is crucial to enable holistic care.

In this study, there is a statistically significant negative correlation between all the QOL domains and depression. Research consistently demonstrates a negative correlation between depressive symptoms and various facets of an individual’s QOL (physical, psychological, social and environmental domains).^37^ The observed inverse association between pain and depression stems from their bidirectional interactions, where each condition exacerbates the other and collectively reduces QOL.^38^

### Implications (clinical and research)

The significant prevalence of depression among cancer patients (35.8%) contrasted with the low rate of reported prior mental illness (1%), which highlights the need for routine screening for depressive symptoms. Increasing mental health awareness can facilitate early identification and management, ultimately enhancing patient care. Integrating mental health services, such as counselling and psychiatric support, into cancer care is essential for improving treatment outcomes.

The link between pain and depression highlights the importance of comprehensive pain management strategies, as prioritising effective pain relief interventions can improve QOL and possibly improve depressive symptoms.

This study paves the way for further research that needs to be done in depression among cancer patients. It is crucial to adopt a longitudinal design to examine the causal relationships between pain, depression and QOL over time, which will help in developing effective and efficient interventions. Furthermore, beyond establishing causality, it is essential to evaluate the effectiveness of both existing and future interventions to improve the management of patients facing both depression and cancer.

### Study limitations

There was only one validated tool used in this study (PHQ-9).^17^ Although the WHO-QOL-BREF has been used in similar settings, little is known about the psychometric properties (validity and reliability) and the cultural sensitivity of the domains included in the instruments.^27^ The stage of cancer was not assessed because majority of participants were unaware of their staging, and this was a self-administered questionnaire, which may impact physical abilities, mental health and QOL. The in-patients had a slower turnover as many of the stable patients travelled from distant areas around the country in order to receive treatment and thus had long stays in the hospital. As a result, it was difficult to get a good in-patient representation in this study because of low turnover. Causality between pain, depression and QOL could not be determined because of the cross-sectional design of the study.^17^

This study has notable strengths, including being the first to quantify depression and QOL specifically in cancer patients in Botswana, with the potential public health benefit of making the case for incorporating mental health screening, psychosocial support and pain control into Botswana’s oncology care. Another strength is its use of a locally validated measure of depression, the PHQ-9, thus strengthening the validity of the findings.

## Conclusion

The results of this study reveal a high prevalence of depression among oncology patients at Princess Marina Hospital. The link between depression and reduced QOL in various areas – physical, psychological, social and environmental – underscores the significant role mental health plays in the overall well-being of cancer patients.

Addressing the dual challenges of cancer and depression is crucial for enhancing the QOL of oncology patients in Botswana. By recognising and treating depression as a critical component of cancer care, healthcare providers can mitigate its detrimental effects, fostering a more holistic approach to patient management in oncology settings. In Botswana, PHQ-9 screening should be a routine, standard part of oncology care.
